# Teacher behavior-related object detection with an edge-enhanced and dynamic multi-scale network toward metaverse-oriented educational scenarios

**DOI:** 10.1038/s41598-026-59541-4

**Published:** 2026-06-25

**Authors:** Jifang Sun, Chengbo Xu, Fang Xie

**Affiliations:** College of Electrical and Mechanical Engineering, Jilin University of Architecture and Technology, Changchun, 130114 Jilin China

**Keywords:** Metaverse education, Teacher behavior detection, Object detection, YOLOv8, Edge-enhanced feature extraction, Dynamic feature fusion, Bounding box regression, Engineering, Mathematics and computing

## Abstract

Teacher behavior analysis is important for understanding instructional patterns and can provide perception support for metaverse-oriented educational environments. This paper focuses on teacher behavior-related object detection in classroom environments and discusses its potential role as a perception module for future metaverse-oriented educational systems. To address challenges such as scale variation, complex backgrounds, and inaccurate localization, an enhanced YOLOv8-based method is proposed. Specifically, an edge-enhanced feature extraction module is introduced to improve boundary representation, and a dynamic inception module is designed to capture multi-scale features. In addition, a Focaler-Shape-IoU loss is adopted to enhance bounding box regression accuracy. These improvements collectively strengthen detection performance. Experiments on a self-collected classroom dataset show that the proposed method achieves competitive results in terms of precision, recall, and mAP@50 compared with several mainstream detectors. The method can effectively handle complex classroom scenarios. Although this work focuses on object detection, it can serve as a foundation for further teacher behavior analysis and may provide foundational perception support for future metaverse-oriented educational applications.

## Introduction

The rapid development of immersive technologies has accelerated the integration of metaverse paradigms into educational environments, giving rise to novel forms of teaching and learning that combine physical and virtual spaces. In this context, teacher behavior detection plays a crucial role as it provides a fundamental bridge between real-world instructional activities and their digital representations^1^. Accurate perception and interpretation of teacher behaviors enable the construction of synchronized virtual classrooms, support data-driven evaluation of teaching quality, and facilitate intelligent tutoring systems capable of real-time feedback. Consequently, behavior-aware object detection can serve as a perception foundation for future metaverse-orienteds education systems^2^.

From a practical perspective, the significance of this problem lies in its potential to transform traditional classroom observation into a continuous, quantitative, and objective process. Conventional teaching evaluation methods often rely on manual observation or post hoc analysis, which are inherently subjective and limited in scalability. By leveraging advanced detection models, teacher actions such as standing, sitting, writing, or engaging with mobile devices can be automatically recognized and temporally tracked^3^. These structured behavioral data can then be utilized to construct comprehensive teaching profiles, identify effective instructional patterns, and support personalized professional development.

Recent advances in artificial intelligence have promoted the development of more robust and adaptive perception frameworks, including spatial-channel collaborative learning, graph interaction modeling, multimodal fusion, reversible guidance strategies, and cyclical knowledge distillation techniques. These approaches improve feature interaction, semantic consistency, and generalization capability under complex scenarios. In addition, fault-tolerant intelligent perception mechanisms have attracted increasing attention for maintaining robustness under noisy inputs, occlusions, and dynamic environments. Such developments provide important technical references for future metaverse-oriented educational perception systems.^4^

Despite its importance, the task presents several technical challenges that distinguish it from standard object detection problems. First, teacher behaviors are inherently fine-grained and context-dependent, often involving subtle differences in posture, hand movement, or object interaction. This requires detection models to go beyond coarse localization and incorporate detailed pose and relational information. Second, classroom environments are characterized by frequent occlusions, cluttered backgrounds, and varying viewpoints, which can significantly degrade detection performance. Third, certain behaviors, such as mobile phone usage or inattentive actions, exhibit strong visual similarity to normal teaching activities, leading to high ambiguity and potential misclassification. Addressing these issues necessitates the integration of multi-modal cues, temporal consistency modeling, and context-aware reasoning mechanisms within the detection framework^5^.

We propose a teacher behavior-related object detection framework for complex classroom scenarios and develop an object detection model based on YOLOv8. Specifically, an edge-enhanced feature extraction (EEFE) module is introduced to improve the model’s sensitivity to object boundaries, thereby enhancing its capability to capture irregular and fine-grained features. In addition, a dynamic inception mixer (DIM) module is designed to achieve adaptive multi-scale feature fusion through a dynamic weighting mechanism, which significantly improves feature representation under complex background conditions. For the loss function, a novel Focaler-Shape-IoU is proposed by integrating the advantages of Focaler-IoU and Shape-IoU. This formulation incorporates geometric information of bounding box shape and scale while emphasizing hard samples, leading to more accurate and robust bounding box regression.

## Related work

### General object detection in educational scenes

Recent advances in object detection and human behavior understanding have significantly improved the feasibility of automated analysis in educational environments. State-of-the-art detectors, particularly those based on the YOLO series and transformer-based architectures, have achieved high accuracy and real-time performance in general object detection tasks. Meanwhile, the integration of human pose estimation and action recognition has enabled more fine-grained interpretation of human activities. In the context of classroom analysis, existing studies have explored teacher and student behavior recognition using combinations of detection, pose estimation, and temporal modeling techniques. However, most approaches remain limited by insufficient sensitivity to subtle behavioral cues, weak robustness under occlusion and complex backgrounds, and a lack of unified frameworks that jointly optimize detection and behavior understanding. Furthermore, the application of these methods in metaverse-based educational scenarios is still in its early stages, with limited work addressing the seamless integration of real-world perception and virtual environment interaction^6,7^.

### Behavior recognition and pose estimation in classrooms

Many scholars have conducted relevant research. Wang et al. employed small-object detection techniques to analyze and evaluate student behaviors in classroom settings. By incorporating label smoothing to reduce sensitivity to noisy annotations, their approach extracts features from preprocessed images and utilizes multi-scale feature fusion to enhance cross-scale semantic representation, aiming to achieve objective and accurate assessment of teaching quality^8^. However, their method focuses primarily on student behaviors and does not address the fine-grained boundary features required for teacher action recognition, where subtle posture differences matter. Xu et al. investigated classroom attention from a first-person perspective and proposed a fusion model integrating gaze tracking and object detection, enabling attention analysis without the need for additional wearable devices^9^. Dang et al. introduced an anchor-free object detector based on center keypoints for behavior recognition in classroom scenarios, where the detection head integrates keypoint heatmaps and a center pooling module to precisely localize object centers^10^. While effective for center localization, this method is less sensitive to object boundaries, which are critical for distinguishing behaviors such as writing versus gesturing. Zhao et al. addressed challenges such as occlusion, pose variation, and scale inconsistency by proposing an advanced single-stage detector that demonstrates strong performance in classroom behavior recognition tasks^11^. Lu et al. further noted that the inherent complexity of classroom environments poses significant challenges to detection performance, and accordingly designed a multi-scale gradient information network that integrates gradient features across scales, achieving real-time end-to-end object detection^12^. Despite achieving real-time performance, their network does not incorporate adaptive weighting across scales, limiting its ability to handle extreme scale variation.

### Limitations and problem formulation

Despite these advancements, existing methods still exhibit limitations in capturing fine-grained behavioral cues, handling complex classroom environments, and achieving robust performance under real-time constraints. In particular, insufficient sensitivity to object boundaries, inadequate multi-scale feature adaptation, and suboptimal bounding box regression remain critical challenges that hinder accurate behavior detection. To address these issues, this study proposes a teacher behavior-related object detection framework for classroom perception tasks, built upon the YOLOv8 framework. By enhancing feature extraction, improving multi-scale representation, and refining localization accuracy through a novel loss formulation, the proposed approach aims to achieve more precise and reliable detection of teacher behaviors, thereby providing a solid foundation for intelligent educational applications.

Dynamic convolution mechanisms, such as CondConv and dynamic filter networks, have demonstrated strong adaptability in handling diverse visual patterns by generating input-dependent convolution kernels. These approaches improve feature representation flexibility while maintaining computational efficiency. Different from directly employing generic dynamic convolution designs, the proposed DIM module focuses on lightweight multi-scale adaptive fusion within the YOLO neck structure for classroom behavior-related object detection.

## Methodology

### System architecture and object detection model

**Fig. 1:**
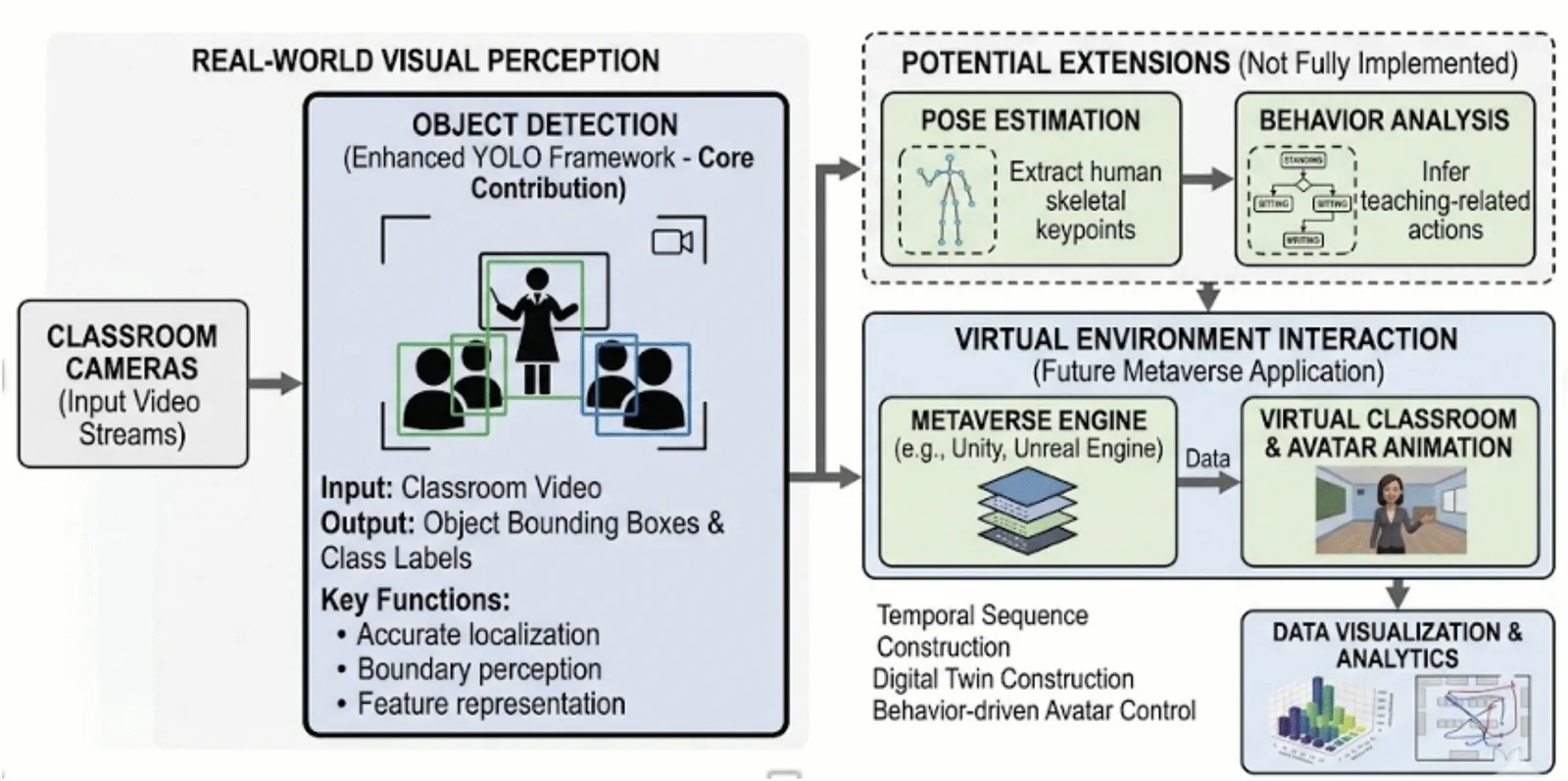
Structure diagram of the system.

The proposed approach targets teacher behavior-related object detection and explores its potential application in metaverse-based educational scenarios. As illustrated in Fig. 1, a conceptual pipeline is presented to describe how real-world visual perception can be connected with virtual environment interaction. In this pipeline, object detection serves as the core component, while subsequent modules such as pose estimation and behavior analysis are considered as potential extensions rather than fully implemented components in this work.

Initially, visual data are captured through classroom cameras, forming the primary input to the system. These video streams are processed by an object detection module based on an enhanced YOLO framework, which is designed to accurately localize key entities such as teachers and relevant objects. The detection stage focuses on improving boundary perception and feature representation, enabling robust performance under complex classroom conditions. This module constitutes the main contribution of this paper and provides the foundation for subsequent behavior analysis.

Beyond object detection, a pose estimation module can be incorporated to extract human skeletal keypoints, which may provide additional structural information about the teacher’s body configuration. Such information could help extend the system from coarse localization to more fine-grained motion understanding. Furthermore, the combination of detection results and pose features could be utilized by a behavior analysis module to infer teaching-related actions, such as standing, sitting, or writing. These components are not the primary focus of this study but represent feasible directions for system extension.

In future metaverse-oriented systems, the detected object and behavior information could potentially be integrated with virtual environment engines into temporal sequences, forming a structured representation of teacher activities over time. This representation may serve as an interface between physical classroom observations and virtual environments. In metaverse-based educational scenarios, such data could be transmitted to a virtual engine (e.g., Unity) to support the construction of a digital classroom and the animation of virtual avatars driven by detected behaviors.

In addition, the generated behavior-related information could be used for visualization purposes, such as activity distribution analysis or trajectory representation, which may assist in understanding teaching patterns. Overall, the presented pipeline illustrates how the proposed detection method can function as a perception module and provide a basis for future integration with behavior analysis and metaverse interaction systems. The system architecture diagram is shown in Fig. 1. It should be noted that the current study mainly focuses on two-dimensional visual perception and object detection, while higher-level functions such as 3D scene reconstruction, digital twin synchronization, and immersive interaction are beyond the scope of this work.

As shown in Fig. 1, we illustrate a conceptual framework for teacher behavior analysis in metaverse scenarios. YOLOv8 is a state-of-the-art single-stage object detection pipeline that follows a decoupled architecture consisting of a backbone, a neck, and a detection head. The backbone is responsible for hierarchical feature extraction from input images, typically employing convolutional layers with cross-stage partial connections to balance computational efficiency and representational capacity. The neck adopts a feature pyramid structure, such as PAN-FPN^13^, to aggregate multi-scale features and enhance the detection of objects at different resolutions. The detection head is designed in a decoupled manner, separating classification and regression tasks to improve convergence and localization accuracy. Additionally, YOLOv8 adopts an anchor-free paradigm, which simplifies the detection process and enhances generalization performance across varying object scales.

Building upon this architecture, an Edge-Enhanced Feature Extraction (EEFE) module is introduced into the backbone to improve the model’s sensitivity to object boundaries. By explicitly emphasizing edge-related information during feature extraction, the proposed module can enable the network to capture fine-grained structural details, thereby enhancing its ability to represent irregular and complex targets. This is particularly beneficial in scenarios where subtle boundary variations are critical for distinguishing different behaviors.

In the neck network, a dynamic inception mixer (DIM) module is designed to strengthen multi-scale feature interaction. Through a dynamic weighting mechanism, the module adaptively fuses features from different receptive fields, allowing the network to selectively emphasize the most informative scale-specific features under varying scene conditions. This design significantly improves the robustness of feature representation in complex backgrounds, where objects may exhibit large variations in size and appearance.

For the loss function, a novel Focaler-Shape-IoU is proposed by combining the strengths of Focaler-IoU and Shape-IoU. This formulation incorporates both the geometric shape and scale information of bounding boxes while introducing a focusing mechanism to prioritize hard samples during training. As a result, the proposed loss enhances the precision and stability of bounding box regression, leading to more accurate localization performance, particularly in challenging detection scenarios.

### Edge-enhanced feature extraction module

**Fig. 2:**
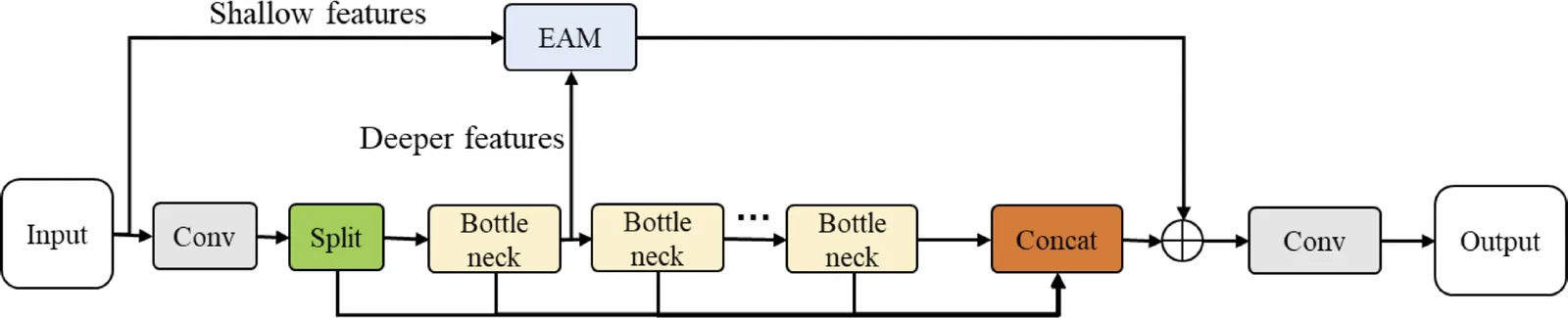
EEFE module structure.

The original YOLOv8 model struggles to accurately capture object boundary information, leading to suboptimal performance when dealing with targets of complex shapes. To address this limitation, an edge-aware enhancement structure based on the C2f framework is designed, an edge-enhanced feature extraction (EEFE) module is proposed to replace the C2f structure in the YOLOv8 backbone, as illustrated in Fig. 2. Unlike existing approaches that simply stack edge enhancement techniques, the proposed module deeply integrates the C2f structure with an edge-aware module (EAM). This design maintains a compact architecture while enabling more effective integration of global semantic information and local edge details, thereby improving the model’s sensitivity to object boundaries and enhancing detection performance for complex targets.

Specifically, the input features are first processed by a *1× 1* convolution for channel reduction, which decreases computational cost while strengthening feature representation. The reduced features are then split into two branches. One branch is directly preserved as a skip connection, while the other is fed into multiple bottleneck modules to progressively extract deeper features. The extracted features are concatenated with the skip connection along the channel dimension, facilitating the fusion of multi-level representations. Subsequently, the fused features are passed through another *1× 1* convolution for channel integration, and are combined with edge features generated by the EAM through element-wise addition, achieving a balanced integration of global semantics and local edge information to produce an optimized feature representation.

**Fig. 3:**
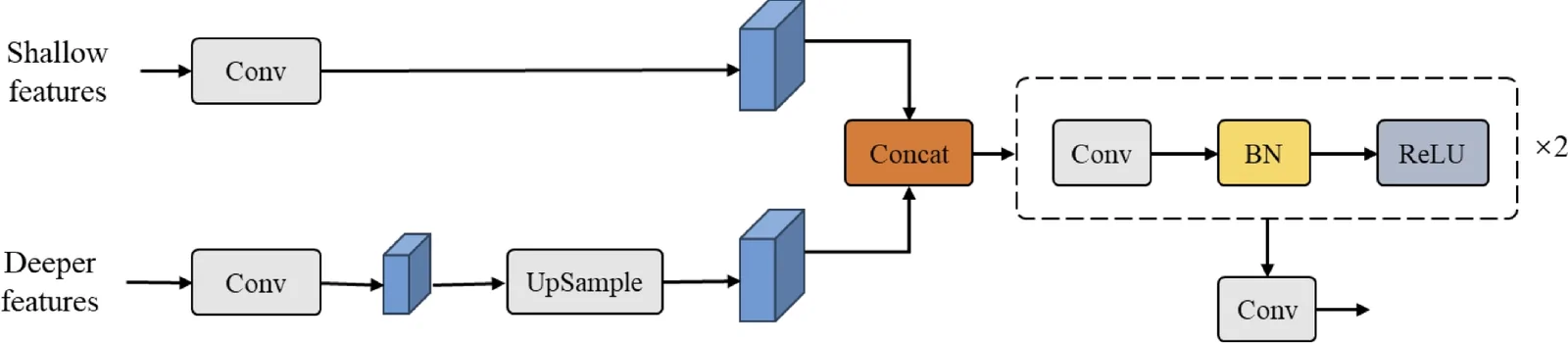
Structure diagram of EAM.

The structure of the EAM is shown in Fig. 3. The module takes two sources of input features: shallow features from the original input and deeper features from the output of the first bottleneck in the EEFE forward path. First, the EAM applies channel compression to the input features, followed by spatial alignment of the backbone features using bilinear interpolation to ensure consistency between feature maps. The aligned features are then concatenated along the channel dimension. The fused features are further refined through two successive *3× 3* convolutions, batch normalization (BN), and ReLU activation, enhancing the representation of edge information. Notably, the output of the first bottleneck is selected as the deep feature input because it has already undergone initial convolution and nonlinear transformation, providing stronger representational capacity. Compared to deeper bottleneck outputs, it preserves higher spatial resolution and achieves better alignment with shallow features, thereby improving edge representation while reducing information loss caused by feature map downsampling. This design effectively balances edge enhancement and semantic understanding.

### Dynamic inception mixer module

**Fig. 4:**
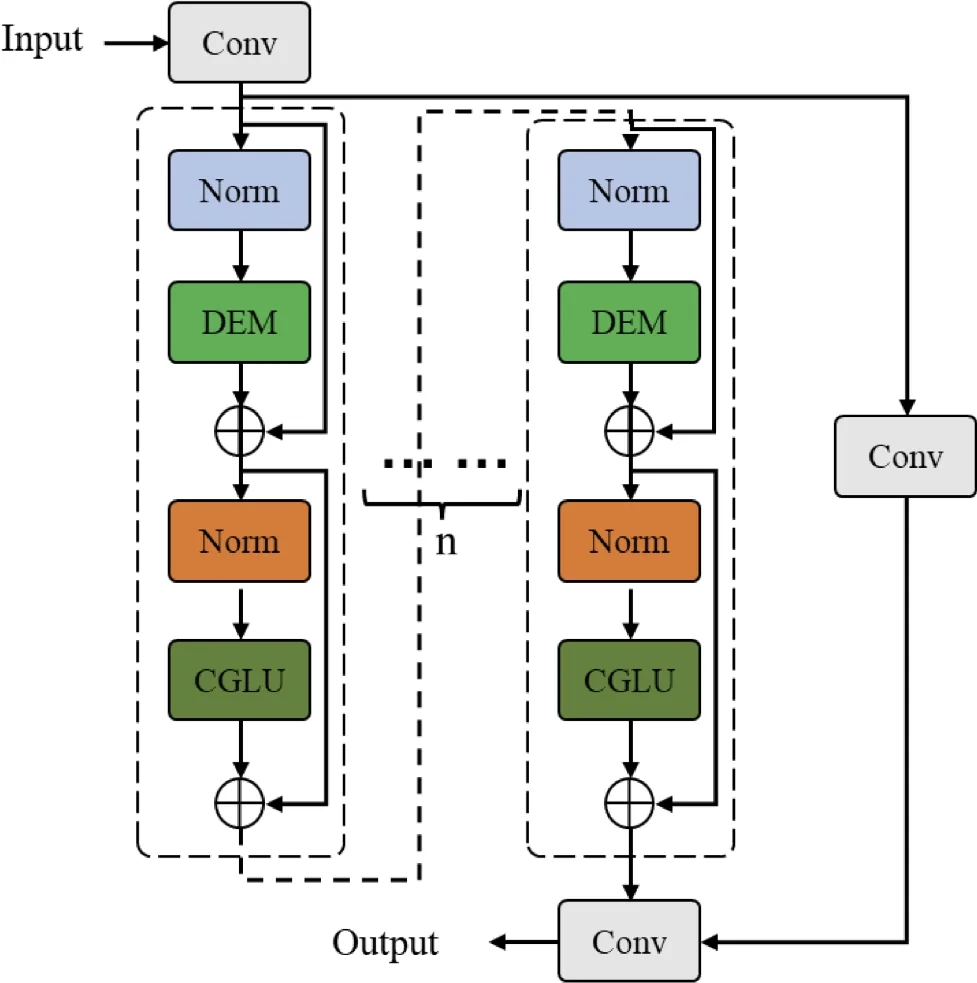
Structure diagram of DIM.

In traditional convolutional neural networks, convolution kernels are typically fixed and static, which imposes certain limitations when handling complex inputs with multi-scale characteristics and high variability. To address this issue, a dynamic inception mixer (DIM) module is proposed, as illustrated in Fig. 4. This module is designed to provide a flexible and efficient convolutional architecture and is embedded into the neck network of the model. The DIM consists of multiple residual enhanced units (REUs). Within each unit, the feature maps obtained after convolution are combined with the input feature maps through residual addition, ensuring effective propagation of both low-level and high-level features. This design not only enables the model to fully exploit multi-level information but also alleviates problems such as gradient vanishing and feature degradation caused by increasing network depth.

The dynamic feature mixer (DFM) module achieves multi-scale feature extraction by combining multiple dynamic depth-wise convolution (DDC) modules with different kernel sizes. The input channels are divided into several groups, each processed by a DDC module, and the resulting features are fused through a *1× 1* convolution. This design allows the model to capture features at different scales and orientations, thereby enhancing its adaptability to diverse target characteristics.

The adaptability of DIM originates from the DDC mechanism, which assigns adaptive weights to each convolution kernel, enabling the network to adjust kernel utilization according to the characteristics of the input feature maps. This mechanism breaks the limitation of traditional fixed convolution kernels, allowing the convolution operation to dynamically adapt during each forward pass and effectively capture multi-scale and diverse feature information.

Specifically, the DDC mechanism first compresses the spatial dimensions of the input feature map to *1 × 1* to extract global contextual information. It then generates weights for three convolutional branches, with the output channel dimension set to three. These weights are normalized into a probability distribution to ensure that their sum equals one. During forward propagation, the input feature map (x) is processed through the three convolutional branches, and the outputs are aggregated via weighted summation according to the learned weights, producing a fused feature map. The resulting feature map is subsequently processed by batch normalization (BN) and a CGLU activation function to produce the final output:

$$\begin{aligned} \textbf{y}=CGLU(BN(\sum _{i=0}^{2} w_i\cdot \mathrm{\operatorname {Conv}}_i(\textbf{x}))) \end{aligned}$$

where $w_i$ denotes the dynamic weight, and $\mathrm{\operatorname {Conv}}_i(x)$ represents the output of the i-th convolutional branch. The CGLU activation function is employed to split the input features and perform gated selection before producing the output, which can be expressed as follows:

$$\begin{aligned} CGLU(\boldsymbol{j})=(\boldsymbol{j}_{:,:,:\frac{d}{2}})\times \sigma (\boldsymbol{j}_{:,:,\frac{d}{2}:}) \end{aligned}$$

where $j_{:,:,:d/2}$ denotes the first half of the last dimension of the input, $j_{:,:,d/2:}$ denotes the second half of the last dimension, and *σ* represents the Sigmoid function.

Unlike generic dynamic filter generation approaches, the proposed DIM emphasizes lightweight multi-branch receptive field interaction and adaptive feature fusion for classroom scenes with large-scale variation and occlusion.

### Focaler-Shape-IoU

In the field of object detection, the accuracy of bounding box regression is critical to the overall performance of detection models. Common regression methods, such as GIoU and DIoU, typically do not account for the varying difficulty levels of different samples and often ignore the intrinsic shape and scale of the bounding boxes . To address these limitations, this paper proposes an improved loss function, termed Focaler-Shape-IoU, which integrates the advantages of Focaler-IoU and Shape-IoU^14^ to enhance regression accuracy, enable adaptive focusing on samples of varying difficulty, and improve overall model performance.

**Fig. 5:**
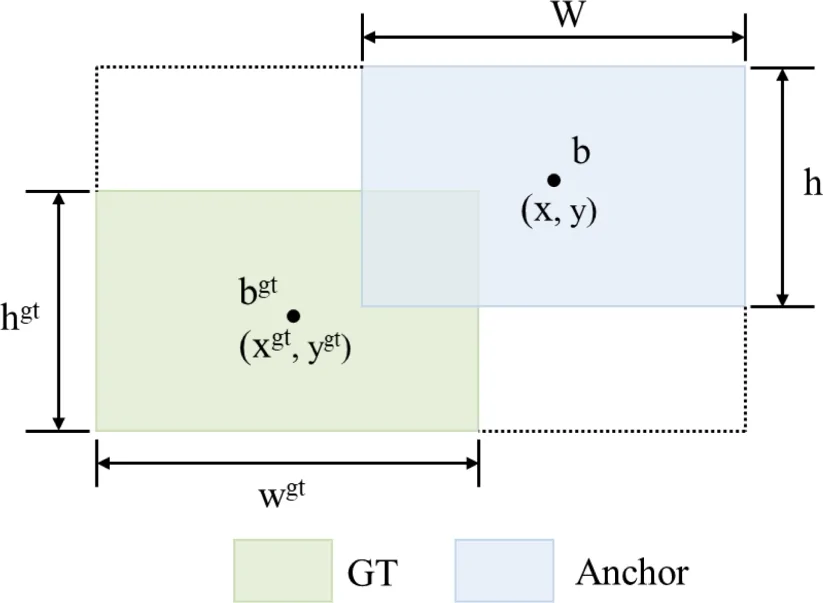
Focaler-Shape-IoU definition diagram.

The Focaler-IoU method reconstructs the original IoU loss through linear interval mapping, allowing the model to adaptively focus on different regression samples across various detection tasks. This approach improves the handling of hard samples and enhances detection performance. Meanwhile, Shape-IoU incorporates geometric properties such as the shape and scale of bounding boxes, thereby improving regression precision. By applying the Focaler-IoU strategy to Shape-IoU, the proposed Focaler-Shape-IoU loss is obtained, replacing the conventional GIoU loss. This loss function adaptively adjusts according to sample difficulty and bounding box geometry, enabling more precise optimization of bounding box regression. As illustrated in Fig. 5, GT and Anchor denote the ground-truth box and the predicted bounding box, respectively.

The calculation formula is:

$$\begin{aligned} IOU^{focaler}=\left\{ \begin{matrix}0,IOU<d& \\ \frac{IOU-d}{u-d},d\leqslant I O U\leqslant u& \\ 1,IOU>u& \end{matrix}\right. \end{aligned}$$

$$\begin{aligned} w_0=\frac{2\times (w^{gt})^{scale}}{(w^{gt})^{scale}+(h^{gt})^{scale}} \end{aligned}$$

$$\begin{aligned} h_0=\frac{2\times (h^{gt})^{scale}}{(w^{gt})^{scale}+(h^{gt})^{scale}} \end{aligned}$$

$$\begin{aligned} d^{shape}=h_0\times \frac{(x_c-x_c^{gt})^2}{c^2}+w_0\times \frac{(y_c-y_c^{gt})^2}{c^2} \end{aligned}$$

$$\begin{aligned} \Omega ^{shape}=\sum _{t=w,h}(1-\operatorname {e}^{-w_t})^\theta ,\theta =4 \end{aligned}$$

$$\begin{aligned} \left\{ \begin{matrix}h_h=h_0\times \frac{|w-w^{gt}|}{max(w,w^{gt})}& \\ & \\ w_w=w_0\times \frac{|h-h^{gt}|}{max(h,h^{gt})}& \end{matrix}\right. \end{aligned}$$

where *scale* denotes a scaling factor related to the size of objects in the dataset, and $w_0$ and $h_0$ represent the weighting coefficients in the horizontal and vertical directions, respectively, which are associated with the shape of the ground-truth (GT) bounding box. Accordingly, the bounding box regression loss is defined as follows:

$$\begin{aligned} Loss=1-IOU^{focaler}+d^{shape}+0.5\times \Omega ^{shape} \end{aligned}$$

## Experiments and analysis

### Datasets and experimental platform

This study employs the teacher behavior subset of the publicly available student classroom behavior (SCB) dataset. The dataset is collected from real-world classroom teaching scenarios, capturing diverse instructional activities under varying lighting conditions, viewpoints, and background complexities. The dataset consists of 8,984 training images, 1620 validation images, and 1620 test images. Each image is annotated with multiple categories related to teacher behaviors and classroom contexts, including guide, answer, on-stage interaction, blackboard-writing, teacher, stand, screen, and blackboard. These annotations provide both behavioral and environmental information, enabling comprehensive evaluation of behavior-aware object detection models.

All experiments were conducted in a deep learning environment based on Python and PyTorch. Model training and evaluation were performed on a workstation equipped with an NVIDIA GeForce RTX 5060 Ti GPU with 16 GB of memory, which provided sufficient computational resources for the proposed detection framework. During training, the number of epochs was set to 300, and the batch size was fixed at 16 to achieve a balance between convergence stability and hardware efficiency. To ensure reproducibility, all experiments were conducted with a fixed random seed and a fixed train, validation and test split.

### Evaluation metrics

To comprehensively evaluate the performance of the proposed model, several widely adopted metrics in object detection are employed, including precision (P), recall (R), mean average precision at an IoU threshold of 0.5 (mAP@50), as well as model complexity indicators such as the number of parameters (Params) and computational cost measured in GFLOPs.

Precision (P, %) reflects the proportion of correctly predicted positive samples among all predicted positives, indicating the model’s ability to avoid false detections. Recall (R, %) measures the proportion of correctly detected targets among all ground-truth instances, representing the model’s capability to detect all relevant objects. The mAP@50 (%) is a comprehensive metric that evaluates detection performance by averaging the precision-recall curves across all categories at an intersection over union (IoU) threshold of 0.5, providing an overall assessment of both localization and classification accuracy. The evaluation indicators are calculated as follows:

$$\begin{aligned} P=\frac{TP}{TP+FP} \end{aligned}$$

$$\begin{aligned} R=\frac{\operatorname {TP}}{TP+FN} \end{aligned}$$

$$\begin{aligned} mAP=\frac{1}{n}\sum _{i=1}^{n}{P_i^{IoU=0.5}(R_i^{IoU=0.5})} \end{aligned}$$

In addition to accuracy-related metrics, model efficiency is evaluated through Params (M), which denotes the total number of trainable parameters in millions, and GFLOPs (G), which represent the number of floating-point operations required for a single forward pass. These metrics are essential for assessing the trade-off between detection performance and computational complexity, particularly in resource-constrained applications. GFLOPs is adopted here as a hardware-independent efficiency measure, since metrics such as frames per second (FPS) are sensitive to the specific GPU platform, software framework version, and inference optimization techniques, which limits their comparability across studies. Given that the proposed model introduces only a modest increase in GFLOPs compared with the baseline YOLOv8n–a detector widely recognized for real-time capability–the inference speed is expected to remain within a practical range. Comprehensive latency benchmarking across diverse deployment environments is deferred to future engineering-oriented work.

### Ablation experiments

To verify the effectiveness of the proposed components, namely the edge-enhanced feature extraction (EEFE) module, the dynamic inception mixer (DIM) module, and the Focaler-Shape-IoU loss, ablation experiments were conducted on the constructed teacher behavior dataset. By incrementally introducing each component into the baseline YOLOv8n framework, the individual and combined contributions of these modules to detection performance were systematically evaluated. This experimental design enables a clear analysis of how boundary-aware feature enhancement, adaptive multi-scale feature fusion, and improved bounding box regression jointly influence the overall accuracy and robustness of the model, thereby demonstrating the rationality and effectiveness of the proposed improvements.

The ablation results in Table 1 demonstrate the effectiveness of the proposed modules in improving detection performance. Starting from the baseline model (Model A), which achieves 88.3% precision, 86.3% recall, and 85.2% mAP@50, the introduction of the EEFE module (Model B) leads to consistent improvements across all metrics. Specifically, precision increases to 89.9%, recall to 88.2%, and mAP@50 to 86.8%, indicating that enhancing edge-aware feature extraction effectively improves the model’s ability to capture boundary information and detect fine-grained behaviors.

**Table 1:** Overall ablation results of EEFE, DIM, and Focaler-Shape-IoU.

Model	EEFE	DIM	Focaler-Shape-IoU	p (%)	R (%)	mAP@50 (%)	Params (M)	GFLOPs (G)
A	×	×	×	88.3	86.3	85.2	3.0	5.8
B	✓	×	×	89.9	88.2	86.8	3.5	6.4
C	✓	✓	×	90.2	89.4	88.1	4.1	6.9
D	✓	✓	✓	90.8	89.2	89.1	4.1	7.0

When both EEFE and DIM are incorporated (Model C), further performance gains are observed. The mAP@50 increases significantly to 88.1%, along with improvements in precision (90.2%) and recall (89.4%). This demonstrates that the DIM module contributes to more robust multi-scale feature representation, enabling the model to better handle variations in object size and complex background conditions. The combined use of EEFE and DIM shows a clear complementary effect, enhancing both localization and classification capabilities.

Finally, with the addition of the proposed Focaler-Shape-IoU loss (Model D), the model achieves the highest overall performance, reaching 90.8% Precision and 89.1% mAP@50. Although Recall shows a slight decrease compared to Model C, the overall detection quality, particularly in terms of localization accuracy, is further improved. This indicates that the proposed loss function effectively refines bounding box regression by incorporating shape and scale information while focusing on hard samples.

In terms of model complexity, the progressive integration of the modules leads to moderate increases in parameters and computational cost, from 3.0M parameters and 5.8 GFLOPs in the baseline to 4.1M and 7.0 GFLOPs in the final model. However, considering the notable improvements in detection performance, the trade-off between accuracy and efficiency remains acceptable. Overall, the ablation study verifies that each proposed component contributes positively to the model, and their combination yields the best performance in teacher behavior detection.

To further investigate the effectiveness of the EEFE module when applied at different locations within the network, comparative experiments were conducted by integrating EEFE into the backbone, the neck, and both simultaneously. This experimental design aims to analyze how edge-enhanced feature extraction influences feature representation at different stages of the model, and to determine the most suitable insertion position for maximizing detection performance. The experimental results are presented in Table 2.

**Table 2:** Ablation results of EEFE inserted at different network positions.

Model	P (%)	R (%)	mAP@50 (%)
base	88.3	86.3	85.2
+EEFE(Backbone)	89.9	88.2	86.8
+EEFE(Neck)	89.5	88.3	85.9
+EEFE(Backbone, Neck)	89.2	88.6	86.1

The results demonstrate that the placement of the EEFE module has a significant impact on detection performance. Compared with the baseline model, introducing EEFE into the backbone yields the most notable improvement, increasing precision from 88.3 to 89.9%, recall from 86.3 to 88.2%, and mAP@50 from 85.2 to 86.8%. This indicates that enhancing edge-aware feature extraction at the early stage of feature learning is highly effective, as it allows the model to better capture boundary and structural information that propagates through subsequent layers.

When EEFE is applied only in the neck, the improvement is less pronounced. Although recall slightly increases to 88.3%, the mAP@50 drops to 85.9%, suggesting that introducing edge enhancement at higher-level feature fusion stages may disrupt the original feature distribution or lead to redundancy, thereby limiting its effectiveness. This implies that edge information is more beneficial when integrated during low-level feature extraction rather than at later stages.

When EEFE is simultaneously applied to both the backbone and neck, the model achieves further improvement in Recall (88.6%), but the overall mAP@50 (86.1%) remains lower than using EEFE in the backbone alone. This suggests that excessive or redundant edge enhancement across multiple stages may introduce feature interference, reducing the overall detection performance.

Overall, the results indicate that incorporating the EEFE module into the backbone is the most effective strategy, as it provides a better balance between precision and feature representation, while avoiding unnecessary complexity or redundancy in later stages of the network.

### Comparative experiments of different models

To further evaluate the effectiveness of the proposed method, comparative experiments were conducted against several representative object detection models, including faster R-CNN^15^, SSD^16^, YOLOv5n^17^, YOLOv7-tiny^18^, YOLOv8n^19^, YOLOv10n^20^, RT-DETR^21^, and Deformable DETR^22^. These models cover both traditional two-stage detectors, lightweight one-stage detectors, and recent transformer-based approaches, providing a comprehensive benchmark for performance comparison. The experimental results are presented in Table 3, where the proposed method is compared in terms of detection accuracy and computational efficiency, demonstrating its advantages in teacher behavior detection tasks.

**Table 3:** Comparison of experimental results of different models.

Model	P (%)	R (%)	mAP@50 (%)	Params (M)	GFLOPs (G)
Faster R-CNN	73.6	72.3	72.5	45.2	131.0
SSD	72.5	71.9	70.2	25.3	56.2
YOLOv5n	85.2	84.5	83.5	2.5	5.2
YOLOv7-tiny	86.8	85.2	83.7	6.2	5.6
YOLOv8n	88.3	86.3	85.2	3.0	5.8
YOLOv10n	86.7	85.2	84.5	2.7	6.5
Deformable DETR	86.6	87.2	84.0	19.9	80.5
RT-DETR	87.2	88.5	86.5	40.5	89.8
Ours	90.8	89.2	89.1	4.1	7.0

The comparative results demonstrate that the proposed method achieves superior performance across both accuracy and efficiency metrics when evaluated against mainstream object detection models. Traditional two-stage detectors such as faster R-CNN exhibit relatively low performance, with a mAP@50 of 72.5%, while also incurring extremely high computational costs (131.0 GFLOPs) and parameter size (45.2M), indicating limited suitability for real-time classroom scenarios. Similarly, SSD shows even lower accuracy, further highlighting the limitations of conventional approaches in handling complex and fine-grained behavior detection tasks.

Among one-stage detectors, lightweight models such as YOLOv5n, YOLOv7-tiny, and YOLOv10n achieve a better balance between efficiency and accuracy. However, their mAP@50 values remain below 85%, suggesting insufficient capability in capturing subtle behavioral differences and handling complex backgrounds. YOLOv8n improves upon these models, reaching 85.2% mAP@50, demonstrating stronger feature representation and detection performance, yet still leaving room for further enhancement.

Transformer-based methods, including RT-DETR and deformable DETR, show competitive performance in terms of recall, with RT-DETR achieving 88.5%. However, these models come with significantly higher computational overhead and large parameter sizes, which limit their practicality for real-time applications in metaverse-based educational systems. Moreover, their mAP@50 performance does not surpass the proposed method.

In contrast, the proposed model achieves the best overall performance, with 90.8% precision, 89.2% recall, and 89.1% mAP@50, outperforming all compared methods. Notably, this improvement is achieved with only 4.1M parameters and 7.0 GFLOPs, maintaining a lightweight structure while delivering substantial gains in detection accuracy. This demonstrates that the integration of EEFE, DIM, and Focaler-Shape-IoU effectively enhances boundary perception, multi-scale feature fusion, and regression accuracy. Overall, the results confirm that the proposed approach achieves an optimal trade-off between accuracy and efficiency, making it well-suited for real-time teacher behavior detection in metaverse-based educational scenarios.

Regarding the generalization capability of the proposed method, it is worth noting that the SCB dataset used in this study is a comprehensive and representative benchmark specifically designed for real-world classroom environments, capturing a wide array of instructional activities under diverse lighting, viewpoints, and background complexities. The three proposed improvements–EEFE, DIM, and Focaler-Shape-IoU–are designed based on fundamental computer vision challenges (boundary representation, multi-scale fusion, and bounding box regression) rather than dataset-specific features. Therefore, these enhancements are expected to be inherently generalizable to other classroom-related datasets. Furthermore, the significant performance gains achieved over established general-purpose detectors (e.g., YOLOv8n, YOLOv10n) on this representative dataset provide strong evidence that the proposed architectural enhancements effectively address the core challenges of teacher behavior detection.

### Visualization experiment

**Fig. 6:**
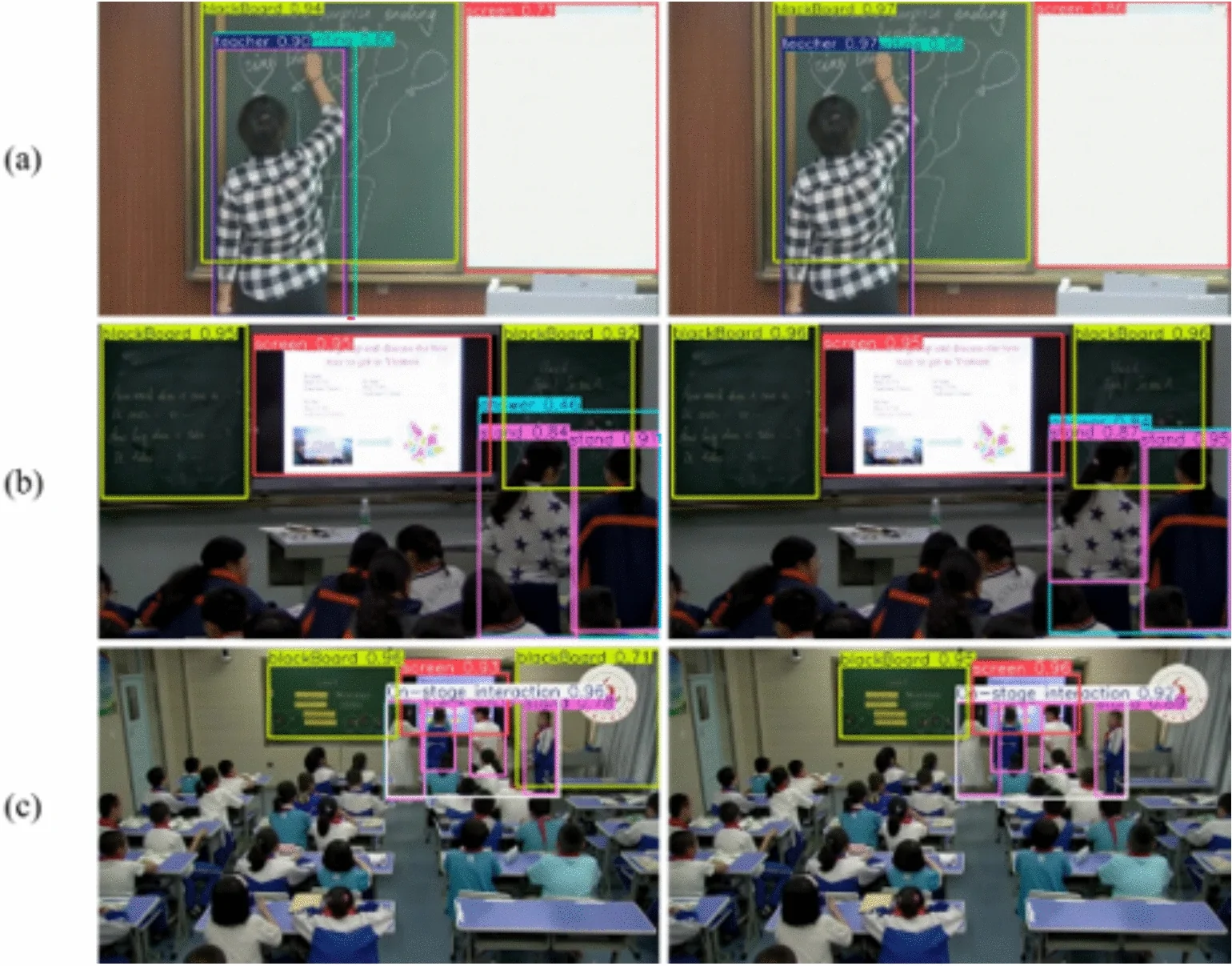
Visualized results.

To further evaluate the practical effectiveness of the proposed model, qualitative visualization experiments were conducted under different classroom scenarios. The results are presented in Fig. 6, where the images on the left correspond to YOLOv8 and those on the right correspond to the proposed model.

As shown in Fig. 6a, the scene depicts a teacher writing on the blackboard. Compared with YOLOv8, the proposed model demonstrates higher detection accuracy for the categories teacher, screen, and blackboard. The predicted bounding boxes are more precise and better aligned with the target objects, indicating improved localization capability in classroom environments.

Figure 6b illustrates a classroom scene under poor lighting conditions. In this challenging environment, the proposed model achieves more accurate detection results for most object categories. In particular, the detection performance for the answer category is significantly improved, with higher confidence and more accurate localization. This suggests that the proposed model possesses stronger feature representation and robustness under low-illumination conditions.

Figure 6c presents a multi-object classroom scenario in which students are giving a presentation at the front of the classroom. While YOLOv8 incorrectly identifies a region on the right side of the image as a blackboard, the proposed model successfully avoids this false detection. This result demonstrates that the proposed model has better discrimination ability and is less susceptible to background interference, thereby exhibiting stronger robustness in complex classroom environments.

Overall, the visual comparison results indicate that the proposed model not only improves detection accuracy but also enhances robustness and generalization capability in complex classroom environments, thereby confirming its superiority over the baseline YOLOv8 model.

## Discussion

Despite the performance gains achieved by the proposed enhanced YOLOv8-based method, several limitations remain that provide opportunities for future research.

First, the current framework relies heavily on high-quality visual data. In practical classroom scenarios, the detection performance may be affected by extreme lighting conditions or severe occlusions (e.g., when the teacher is completely blocked by equipment or students), where visual information alone is insufficient for accurate localization. To address this, future work could explore the integration of different learning paradigms, such as semi-supervised or self-supervised learning, to leverage unlabeled data and improve the model’s generalization capability in diverse environmental conditions.

Furthermore, to enhance the robustness of perception in cluttered and nonstationary environments, the system could be extended from a single-modality visual framework to a multi-modal perception system. While visual sensors provide rich semantic information about behaviors, they are susceptible to line-of-sight obstructions. Integrating complementary sensing technologies, such as device-free localization based on commodity Wi-Fi devices, could provide a more stable foundation for tracking. For instance, recent advances in online spatiotemporal modeling for robust and lightweight device-free localization in nonstationary environments^23^ and techniques for accurate localization in cluttered environments^24^ demonstrate that non-visual signals can maintain high reliability even when visual paths are obscured. Combining these robust localization capabilities with the proposed behavior-related object detection would enable a more resilient perception module for metaverse-oriented educational scenarios, ensuring continuous tracking of teacher activities regardless of visual occlusions.

Finally, while this study focuses on two-dimensional object detection, extending the framework to three-dimensional space or integrating temporal sequence modeling (such as using transformers or RNNs) would allow for a more comprehensive analysis of behavior dynamics over time, moving from static object detection toward a full-scale behavior understanding system.

## Conclusion

This paper presents an enhanced YOLOv8-based method for teacher behavior-related object detection in classroom scenarios. To address challenges such as complex backgrounds, scale variations, and inaccurate localization, three complementary improvements are introduced, including the edge-enhanced feature extraction (EEFE) module, the dynamic inception module (DIM), and the Focaler-Shape-IoU loss. These components jointly improve feature representation and bounding box regression, leading to more accurate and robust detection performance. Experimental results on a self-collected classroom dataset demonstrate that the proposed method achieves competitive performance compared with several mainstream object detection models in terms of precision, recall, and mAP@50. The results indicate that the proposed approach can effectively handle challenging classroom environments and improve the detection of teacher-related behaviors. It should be noted that this work primarily focuses on object detection as a foundational task. While a conceptual pipeline involving pose estimation, behavior analysis, and virtual environment interaction is discussed, these components are not fully implemented or evaluated in this study. Instead, the proposed method is intended to serve as a perception module that can support subsequent behavior analysis. In the context of metaverse-based educational applications, the proposed approach has the potential to provide reliable visual perception for virtual classroom systems. Future work will focus on integrating temporal modeling, pose estimation, and behavior recognition to enable more comprehensive teacher behavior understanding and to support interactive virtual environments. Extending the current framework to encompass three-dimensional scene reconstruction, digital twin synchronization, and immersive interaction modeling constitutes an important direction for future work.

## Data Availability

The dataset used in this study is publicly available. We have now clearly stated this in the revised manuscript and provided the direct access link in both the Methods section and the Data Availability section. The dataset can be accessed at:https://github.com/Whiffe/SCB-dataset.
